# Influence of Different Drying Processes on the Chemical and Texture Profile of *Cucurbita maxima* Pulp

**DOI:** 10.3390/foods13040520

**Published:** 2024-02-08

**Authors:** Antonela Ninčević Grassino, Sven Karlović, Lea Šošo, Filip Dujmić, Marija Badanjak Sabolović, Marko Marelja, Mladen Brnčić

**Affiliations:** Faculty of Food Technology and Biotechnology, University of Zagreb, Pierottijeva 6, 10 000 Zagreb, Croatia; aninc@pbf.hr (A.N.G.); skarlovi@pbf.hr (S.K.); leasosso96@gmail.com (L.Š.); filip.dujmic@pbf.unizg.hr (F.D.); mbadanjak@pbf.hr (M.B.S.); mmarelja@pbf.hr (M.M.)

**Keywords:** fresh and dried pumpkin pulp, hot air drying, vacuum drying, conductive drying, ultrasound-assisted extraction, chemical and texture profile

## Abstract

The effects of hot air (HAD), vacuum (VAD) and conductive (CD) drying on the chemical and textural profiles of *Cucurbita maxima* pulp were investigated to find suitable drying conditions to avoid postharvest losses of pumpkin. The results showed that the drying methods had a significant effect (*p* < 0.05) on the chemical and textural profiles of pumpkin pulp. The ash content was lower in VAD (up to 7.65%) than in HAD (up to 9.88%) and CD pulp (up to 9.21%). The samples of HAD, CD and VAD had a higher fat content, up to 3.07, 2.66 and 2.51%, respectively, than fresh pulp (1.55%). The total fibre content is lower for VAD (up to 8.78%) than for HAD (up to 15.43%) and CD pulp (13.94%). HAD pulp at 70 °C (~15.51%) and VAD and CD pulp processed between 50 and 60 °C (~22%) are good sources of protein. HAD and CD pulp at 70 °C and VAD at 50 °C resulted in a high sugar content (up to 83.23%). In addition to drying, the extraction time of 40 min used in ultrasound-assisted extraction is optimal, especially for protein and sugar recovery in dried samples. Drying also led to strong changes in the textural properties of the pulp, so that an excellent dried intermediate product is the one obtained using HAD at a temperature of 70 °C and an airflow of 0.5 m/s.

## 1. Introduction

Pumpkin (*Cucurbita* spp.), which belongs to the gourd family (*Cucurbitaceae*), is an important crop that is cultivated and consumed worldwide for its nutritional and health-promoting properties [1,2,3]. Pumpkin is a rich source of carotenoids, phenolic acids, flavonols, vitamins, polysaccharides and minerals [4,5,6,7,8], which makes it a valuable functional food.

The world production of pumpkin is about 27 million tons, of which China as the main producer accounts for 5.5 tons and Europe for more than 4 million tons [9]. The most commonly grown pumpkin species of commercial importance are *Cucurbita pepo*, *Cucurbita moschata* and *Cucurbita maxima*, whose fruits differ in size, shape and colour. The cracked fruits have yellow-orange flesh, which makes them very attractive to consumers. However, the pumpkin fruits are seasonal and more susceptible to bacterial and fungal attack and colour changes. Therefore, considering the wide production and use of pumpkin (cooked, baked and processed into various products, e.g., pumpkin pie, bread, cookies, cheesecake, desserts, donuts, cereals, ice cream, lasagna dishes, etc.), it is necessary to maintain its nutritional and phytochemical profile over a long period of time. High water content in fresh fruit is associated not only with microbial reactions, but also with chemical and enzymatic reactions that negatively affect product quality and shelf life. To extend the shelf life of pumpkin by reducing water activity to a level that greatly inhibits spoilage, drying is a promising tool.

Drying is an effective preservation technology that not only extends the shelf life of the target product, but also reduces its volume and weight during transportation, which in turn reduces packaging and storage costs. Depending on the mechanism of heat transfer, a distinction is made between direct (convection) drying, indirect or contact (conduction) drying, radiation drying and dielectric or microwave (high-frequency) drying [10,11,12,13].

Direct or convective drying (application of hot air) is the most commonly used technology for the industrial preservation of fruits and vegetables because it involves low operating costs and the process can be easily controlled. However, hot air drying (HAD) can cause various irreversible quality losses in raw materials, such as colour, nutritional value, texture, porosity, etc. Since prolonged contact with atmospheric oxygen at high temperatures can lead to the degradation of valuable components sensitive to oxidation, alternative drying methods have been proposed [14,15]. For example, in vacuum drying (VAD), the contact between the material and oxygen is limited, making this method suitable for oxygen-sensitive materials. Due to the reduced pressure, effective drying can be achieved at low temperature, making this method suitable for heat-sensitive materials.

After drying, the pumpkin can be used to prepare stews and soups, or the dried pumpkin can be processed into flour and used as an ingredient for various pumpkin-based products, such as pumpkin biscuits, cakes, bread, noodles and yogurt [16,17]. Therefore, knowledge of the nutritional value and texture of dried pumpkin pulp would be important information for consumers to encourage consumption of this vegetable in the off-season.

The sensory perception of foodstuffs is of huge importance considering consumers’ demands these days. Particularly, no matter how functional of highly valued produced food commodities could be, if the various sensorial attributes, such as texture, colour and taste, are rather negative, the consumer will probably reject it. Therefore, the texture and its determination after the processing is complete, play a significant role in the embracement of the products [18,19,20]. Moreover, various changes that occur during the drying caused by the water loss fosters significant structural changes in the dried products followed by the different textural properties in comparison with fresh samples [21].

In this research, the conventional extraction procedure to obtain proteins and sugars was used and compared with innovative ultrasound-assisted extraction. As an environmentally friendly technique, ultrasound in general is in wide usage in the overall food industry and covers extraction, homogenization, enzyme inhibition and inactivation, drying and microbe inactivation as well [22,23,24].

Since there are no comprehensive data on the chemical and textural profiles of *Cucurbita* spp. in the literature [3,14,25,26,27,28,29,30,31,32,33,34,35,36] and, in particular, no data on the comparative composition of fresh and dried pumpkin pulp obtained by different drying methods, the present study focuses on the influence of hot air drying (HAD), vacuum drying (VAD) and conductive drying (CD) on the chemical and textural composition of *Cucurbita maxima* pulp. In addition to drying, the present work also provides a solution for the extraction of proteins and sugars from pumpkin fruit pulp samples using ultrasound-assisted extraction (UAE) as a rapid and environmentally friendly technique instead of the conventional one (CE). Thus, the main objective of this work was to determine which of the drying methods used gives the appropriate chemical and textural properties of the dried pulp obtained. The information obtained from this study could thus provide a basis for the selection of drying methods and parameters that can be used for the further production of high-quality dried pumpkin pulp.

## 2. Materials and Methods

### 2.1. Chemicals and Reagents

All reagents, standards and solvents were of analytical grade. Glucose monohydrate and bovine serum albumine used for the determination of sugars and proteins were purchased from Sigma Aldrich (Steinheim, Germany). Petroleum ether, sulfuric acid, copper sulphate, potassium iodide, potassium sodium tartarate and sodium hydroxide were purchased from Kefo (Zagreb, Croatia). Phenol was provided by Acros Organics (Geel, Belgium). Deionized water used for the preparation of reagents, standards and solvents was obtained using the Millipore Milli-Q instrument.

### 2.2. Material Preparation

Fresh pumpkin (*Cucurbita maxima*) was purchased from Exotic king (Šulog, Donja Bistra, Croatia). The fruits were transported to the laboratory, washed with tap water and cut in half lengthwise with a stainless steel knife. After the separations of seeds, fibrous strands and peel, the pulp was cut into thick slices with an adjustable kitchen knife. The pulp was then cut into 3.5 to 0.5 mm thick slices with an adjustable kitchen knife and dried.

### 2.3. Drying Process

Fresh pumpkin pulp of *Cucurbita maxima* was dried using different processing parameters employed in hot air drying (HAD), vacuum drying (VAD) and conductive drying (CD). The designation of the samples based on the applied temperatures, airflow velocities and pressures are shown in Table 1.

#### 2.3.1. Hot Air Drying

Hot air drying was carried out at three different temperatures of 50, 60 and 70 °C and airflow velocities of 1.5, 1.0 and 0.5 m/s using an ARMFIELD UOP8-MKII dryer (Armfield Limited, Ringwood, UK).

Fresh pumpkin pulp slices (500 ± 2.7 g) were spread evenly in a single layer on a perforated tray containing three aluminium trays of size 30 × 25 cm (Silverwood, Birmingham, UK). Before drying, the dryer was switched on for 30 min to achieve steady-state conditions. The trays were placed in the drying chamber, previously heated to achieve steady-state conditions. During the drying process, the mass of the trays with samples was recorded constantly every 2 min. Constant mass was an indicator of the end of the drying process. Then, dried pumpkin slices were removed from the dryer and allowed to cool at room temperature for 15 min. They were then packed in polyethylene bags and stored in a refrigerator (4 ± 1 °C) until further analysis.

#### 2.3.2. Vacuum Drying

Vacuum drying was carried out at three different temperatures of 50, 60 and 70 °C and a vacuum pressure of 100 mbar (Table 1) using a Memmert VO 200 vacuum drying oven with a Memmert PM200 pump module (MEMMERT GmbH + Co. KG, Schwabach, Germany). The preparation of the samples for drying was described by Karlović et al. [37].

#### 2.3.3. Conduction Drying

Conduction drying at an atmospheric pressure of 1000 mbar was carried out at three different temperatures of 50, 60 and 70 °C (Table 1) using a Memmert VO 200 drying oven without Memmert PM200 pump module (MEMMERT GmbH+Co.KG, Schwabach, Germany). The samples were prepared in the same way as the samples for vacuum drying [37].

### 2.4. Chemical Analysis

**Figure 1:**
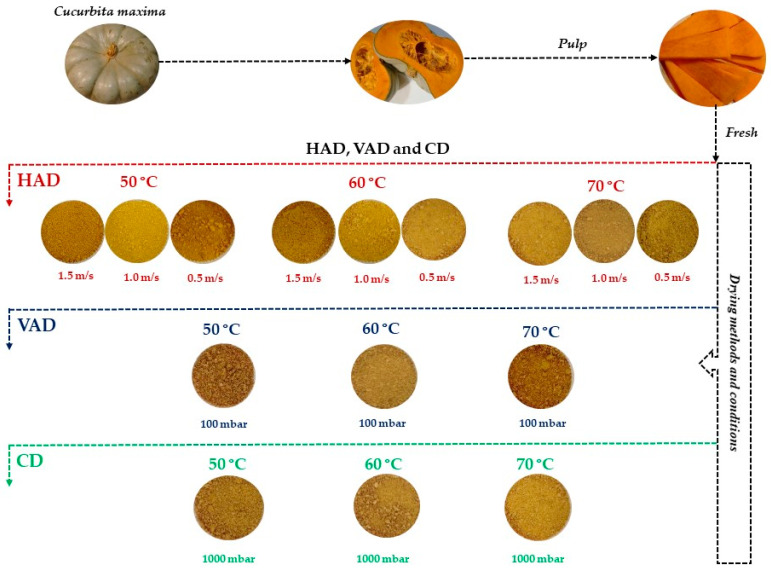
Schematic representation of *Cucurbita maxima* pulp samples after hot air drying (HAD), vacuum drying (VAD) and conductive drying (CD).

Moisture content, total ash, total fat and total fibre content were determined in fresh and dried pumpkin pulp (Figure 1). Prior to analysis, all samples were ground using a laboratory mill. Moisture, ash, fat and fibre were determined according to the Association of Official Analytical Chemists (AOAC) methods [38].

Briefly, moisture content and ash were determined at 105 °C and 600 °C, respectively, to a constant weight in a drying chamber and muffle furnace. Total fat was determined gravimetrically after the dehydrated samples were extracted with petroleum ether in a Soxhlet apparatus for 8 h. Total fibre was determined gravimetrically after the defatted samples were hot digested for 1 h in an aqueous solution of sulfuric acid (*c* = 0.255 mol/L) or an aqueous solution of sodium hydroxide (*c* = 0.313 mol/L) under reflux. After filtration and purification (hot water and ethanol), the insoluble residues were dried (24 h), cooled (1 h) and weighed. They were then incinerated at 600 °C for 4 h.

Except for moisture content, which was expressed as g of moisture per 100 g of sample on a wet basis (wb), all other results were expressed on a dry basis (db) to eliminate the water effect and thus allow a comparison of values between different dried samples (see also protein and sugar analyses). All experiments were performed in triplicate.

### 2.5. Protein and Sugar Extraction

The extraction of proteins and sugars from fresh and dried pumpkin pulp (Figure 1) was performed using UAE at a temperature of 50 °C, a frequency of 37 kHz and a working amplitude of 95% (ultrasonic bath, Elmasonic, Germany). The duration of sonication was 10, 20 and 40 min. To compare the efficiency of UAE, the extraction of proteins and sugars was also performed using conventional extraction (CE) under reflux for 20 and 40 min at 50 °C.

The sample-to-solvent ratio for the extraction of proteins and sugars was 1:30 (*w*/*v*) in CE and UAE. Protein extraction was performed with phosphate buffer (pH = 7.4), while deionized Millipore Milli-Q water was used for sugar extraction. After extraction, the mixture was allowed to cool at room temperature and then filtered. The extracts were collected and stored at −8 °C until further analysis.

### 2.6. Protein Analysis

The total soluble protein content was determined according to the Biuret method with some modifications [39]. In this method, the Biuret reagent was added to each of the pumpkin extracts, i.e., fresh and dried (HAD, VAD and CD) pumpkin pulp according to UAE and CE. The samples were shaken and incubated for 30 min at room temperature in the dark. Then, absorbance was measured at 540 nm using a Perkin Elmer Lambda 25 spectrophotometer (Shelton, MA, USA).

Bovine serum albumin was used as a standard to generate a calibration curve. The linear regression equation with *R*^2^ = 0.9981 was: *A*540 = 0.2215 × (bovine serum albumin)/(mg/mL). Measurements were performed in triplicate, and the results were expressed as g total proteins per 100 g sample on a db.

### 2.7. Sugar Analysis

Total sugars were determined using the phenol-sulfuric acid method [40] with some modifications. In this method, a 5% phenol solution and concentrated sulfuric acid were added to each pumpkin extract, i.e., fresh and dried (HAD, VAD and CD) pumpkin pulp according to UAE and CE. After homogenization, the mixture was heated in a boiling bath for 5 min, cooled in an ice bath and stored in the dark for 30 min. Subsequently, absorbance was measured at 490 nm using a Perkin Elmer, Lambda 25 spectrophotometer (Shelton, MA, USA).

Glucose was used as a standard to generate a calibration curve, and the linear regression equation with *R*^2^ = 0.9991 was *A*492 = 0.0533 × (glucose)/(mg/L). Measurements were performed in triplicate, and the results were expressed as g total glucose per 100 g sample on a db.

### 2.8. Texture Analysis

The texture of fresh and dried (HAD, VAD and CD) pumpkin pulp (Figure 1) was determined using universal testing machines (Stable Micro Systems Texture Analyzer TA.HD.plus, Godalming, UK). Textural profiles were determined by compressing the sample in two consecutive cycles between parallel plates with an 8 mm diameter probe and a 5 s interval between cycles. The textural properties of hardness, elasticity and work performed were calculated using SMS Exponent software (ver. 6.2.2.0, Godalming, UK). Hardness was determined as the maximum force during the first peak on the obtained force-displacement curve. Elasticity was determined as the distance between the start of compression and the first peak, and work performed was calculated as the area under the entire curve.

### 2.9. Statistical Analysis

The results were expressed as mean ± standard deviation of triplicate measurements (*N* = 3). Significant differences (*p* < 0.05) within means were examined using analysis of variance (ANOVA) and Tukey’s test for honestly significant differences (HSD). All tests for data analysis were evaluated using SPSS Statistics software version 26 (IBM, New York, NY, USA).

## 3. Results and Discussion

Pumpkin can be marketed to the consumer in the form of freshly cut pumpkin slices, preserved using an edible coating and vacuum packaging. Pumpkin powders, extracts, isolates and pumpkin derivatives [17] are potential ways to utilize this nutritious fruit. Therefore, the accurate determination of basic nutrients not only in fresh but also in dried pumpkin is important information for the consumer as the demand for reliable food labelling has increased.

In this work, the effects of three different drying methods (HAD, VAD and CD) and drying parameters (Table 1) were investigated to find suitable drying conditions to obtain the best nutrient and texture profiles of the dried pumpkin pulp samples (Figure 1). In this context, moisture content, total ash content, total fat content, total fibre content, total protein content and total sugar content and texture were analyzed and compared with the values of the fresh samples to highlight the efficiency of drying. Due to the lack of drying studies for pumpkin (Table 2), especially VAD and HAD, this study and its obtained results can provide valuable data not only for pumpkin producers but also for consumers.

### 3.1. Moisture Content

**Figure 2:**
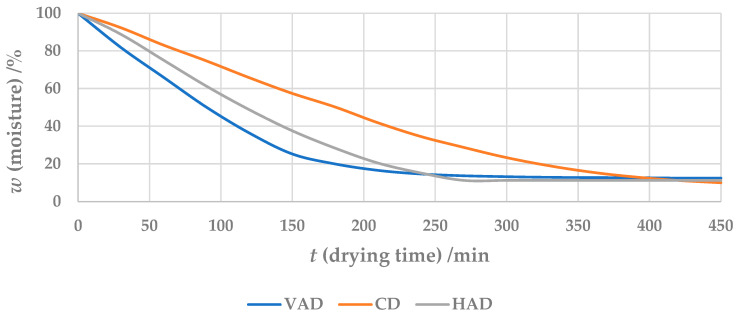
Drying curves according to the drying processes, HAD (60 °C, 1.0 m/s), VAD (60 °C) and CD (60 °C).

Once the pumpkin pulp samples had reached a constant mass, the drying process was terminated. Figure 2 shows an example of the drying kinetics of three drying processes, namely HAD (60 °C, 1.0 m/s), VAD (60 °C) and CD (60 °C). It can be seen that VAD drying is the fastest drying method at the same temperature. This is followed by HAD drying, where the airflow velocity has a significant influence on the drying time. The slowest drying times are achieved with CD technology. As drying is carried out to a constant mass, it can be seen from the drying curves that there are differences in the mass of water removed.

**Figure 3:**
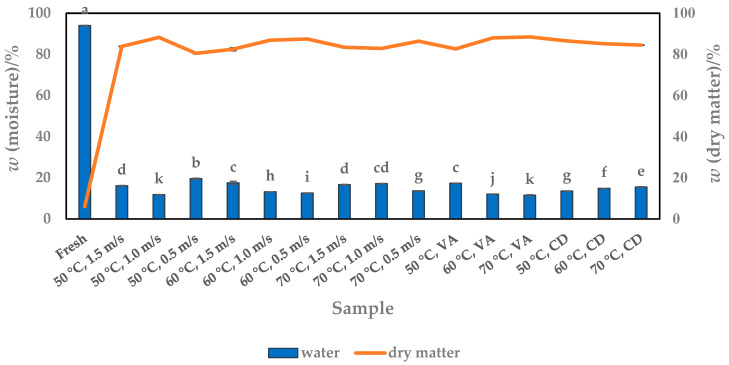
Moisture content versus dry matter content of fresh and dried (HAD, VAD and CD) pumpkin pulp. Values are means ± standard deviations of three (*N* = 3) measurements. Different superscript lowercase letters in the same column indicate significant differences (*p* < 0.05) between drying methods (HAD—hot air drying, VAD—vacuum drying, CD—conductive drying) and conditions.

**Figure 4:**
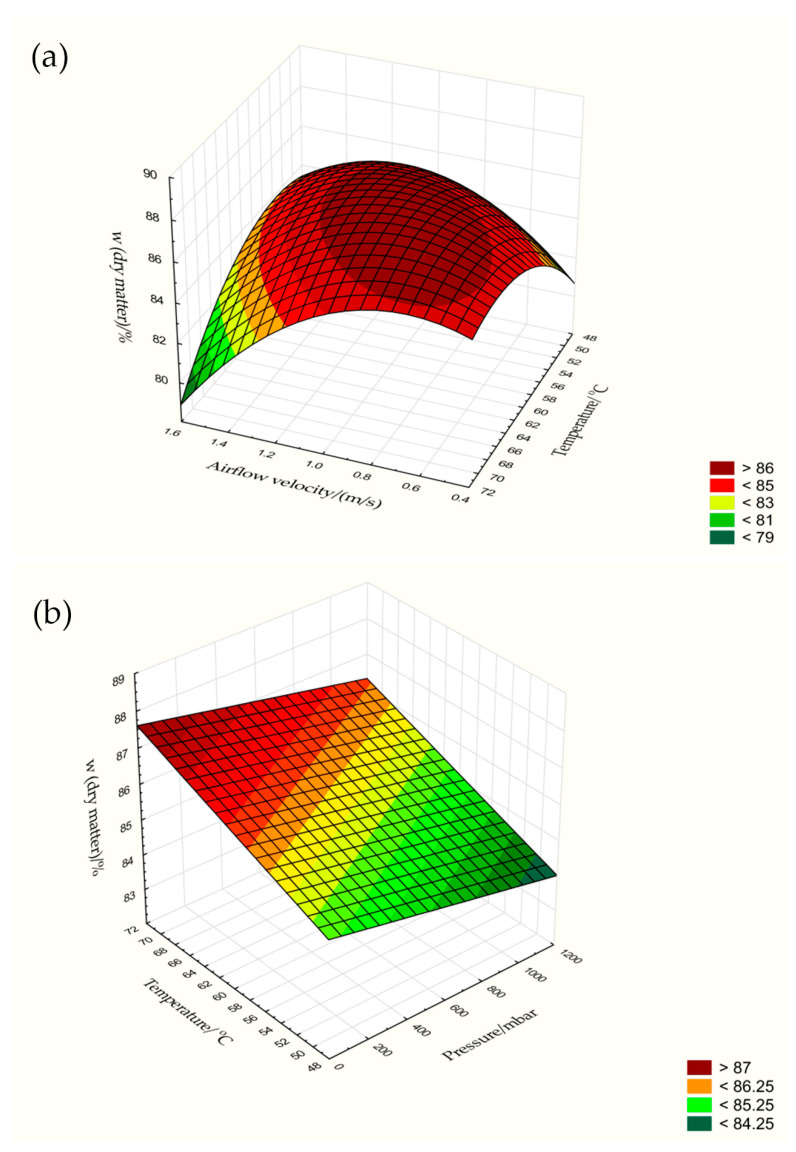
Influence of temperature (50, 60 and 70 °C) and airflow velocity (0.5, 1.0 and 1.5 m/s) (**a**) and temperature (50, 60 and 70 °C) and pressure (100 and 1000 mbar) (**b**) on the dry matter content of pumpkin pulp according to HAD or VAD and CD.

Moisture content or dry matter is one of the most important characteristics of food and is directly related to its quality [15,41]. In this work, it was found that the moisture content of fresh pumpkin was 93.97%, which is close to the results of other works [3,14,25,26,27,28,29,30,31,32,33,34,35,36] with values between 82.10 and 92.34%, depending on the variety of Cuburbita. Drying reduced the moisture content from 93.97% to 11.52%, so that the dry matter content increased from fresh (6.03%) to dried pulp (80.45–88.48%, depending on the HAD, VAD and CD processes). The dry matter content of the HAD pulp ranged from 80.45 to 88.22%, depending on the temperature (50, 60 and 70 °C) and airflow velocities (0.5, 1.0 and 1.5 m/s). These combined parameters had a significant influence (*p* < 0.05) on the dry matter content (Figure 3 and Figure 4a), so that the lowest value of 80.45% was determined for the samples dried at 50 °C and an airflow velocity of 0.5 m/s. A better drying effect is given when the temperature of 50 °C is combined with higher airflow velocities of 1.0 m/s (88.22%) or when temperatures of 60 °C with velocities of 0.5 m/s (87.45%) and 1.0 m/s (86.84%) or 70 °C with a velocity of 0.5 m/s (86.38%) are applied. A significant influence (*p* < 0.05) of temperature on the dry matter content was also demonstrated for the VAD samples, where an increase in temperature at a constant pressure of 100 mbar increased the dry matter content from 82.63 to 88.48% (Figure 3 and Figure 4b). In contrast to VAD, the results of CD showed that increasing the temperature at a constant pressure of 1000 mbar slightly decreased the dry matter content from 86.51 to 84.48% (Figure 4b). We suspect that in addition to the drying conditions and the mechanism influencing the final moisture content, a number of factors also play a role from an analytical point of view. For example, the uniform composition and distribution of water and other analytes in the fresh pumpkin pulp are key factors influencing the final dry matter content. Water can be present as an impurity from the atmosphere or bound as a chemical compound, e.g., as a hydrate. We suspect that the interaction between water and nutrients makes the release of moisture more difficult, and we believe that this is the main reason why the water content in the CD sample dried at 70 °C was slightly lower than in the sample dried at 50 °C, i.e., the interactions of water with other compounds in the pulp are stronger. Especially with solid substances such as pumpkin pulp, which is a complex mixture of different compounds, the water content can vary and depends, among other things, on the humidity, temperature and condition of the sample before and after drying.

From the results, it can be concluded that although HAD, VAD and CD lead to a highly dehydrated pulp (80.45–88.48%), VAD achieves better moisture reduction compared to the other drying methods due to differences in the drying mechanism. In addition to the VAD process, the HAD process also led to better moisture reduction at airflow velocities of 1.0 and 0.5 m/s and temperatures of 50 and 60 °C or 70 °C, so that both processes with these parameters would be suitable for the future drying of pumpkin pulp.

### 3.2. Ash Content

**Figure 5:**
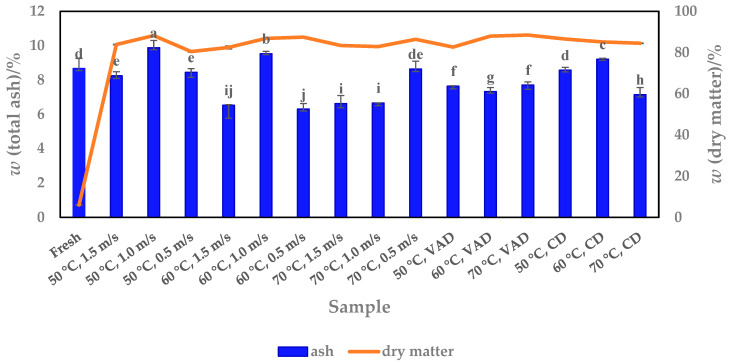
Total ash content versus dry matter content of fresh and dried (HAD, VAD and CD) pumpkin pulp. Values are means ± standard deviations of three (*N* = 3) measurements. Different superscript lowercase letters in the same column indicate significant differences (*p* < 0.05) between drying methods (HAD—hot air drying, VAD—vacuum drying, CD—conductive drying) and conditions.

The total ash content as a measure of the inorganic elements or minerals was determined in fresh and dried pumpkin pulp. It was found that fresh pulp contains 0.54% (wb) ash, which corresponds to 8.67% on a db (Figure 5). A similar ash content of 0.84% (wb) for fresh pulp of *Cucurbita maxima* was determined by Montesano et al. [29], while Umuhozariho et al. [28] and Guine et al. [30] reported values of 3.21% (wb or db is not stated) and 13.99% (db), respectively. The data for fresh pumpkin with the species *Cucurbita moshata* were higher (6.16%, wb) [33] than the values found in this work.

The results for dried samples ranged from 6.31 to 9.88% (db), depending on the drying processes and parameters, which significantly affected the ash content (Figure 5). Some of the HAD samples (50 °C, 1.5 m/s, 50 °C, 0.5 m/s and 70 °C, 0.5 m/s) had similar values (8.25%, 8.45% and 8.64%) to fresh pulp (8.67%), while the 60 °C, 1.0 m/s and 50 °C, 1.0 m/s samples were found to have higher values of 9.53% and 9.88%, respectively, compared to fresh pulp. The other HAD samples, e.g., 60 °C (0.5 and 1.5 m/s) and 70 °C (1.0 and 1.5 m/s), had a lower ash content than fresh pulp, ranging from 6.31 to 6.66%. Apart from the fresh pulp, the HAD pulp results showed that increasing both drying parameters, temperature and airflow velocity, leads to a reduction in ash content. The optimum ash yield is therefore achieved at a temperature of 50 and 60 °C and an air flow velocity of 1.0 m/s. The decreasing effect on ash content (7.32–7.71%) is observed for VAD samples compared to fresh pulp, indicating that this drying method is less efficient than HAD and CD. At 50 °C and 60 °C, the CD samples showed an ash content of 8.57% and 9.21%, respectively, which is very similar or even higher than that of fresh pulp. Therefore, the temperature of 70 °C should no longer be used for CD, as it significantly lowers the ash content (Figure 5). Overall, we suspect that the differences in the results are mainly related to the uniformity of the composition of the pumpkin pulp, i.e., the inner part enclosed near the seeds and the fibre stock may not have the same mineral composition as the part near the peel. Sampling the pumpkin pulp before drying may therefore have an influence on the ash content.

### 3.3. Fat Content

Vegetable oils are an important source of essential fatty acids, and their consumption in the human diet in the ratio between saturated and unsaturated fatty acids has attracted much attention. Nowadays, many studies have been conducted on oil extraction from seeds, including pumpkin varieties and species [42,43,44,45], which have been shown to affect the oil yield. In addition to determining the oils from the seeds, this study also presents the results of fat analyses of fresh and dried pumpkin pulp to determine which drying methods affect the fat value.

**Figure 6:**
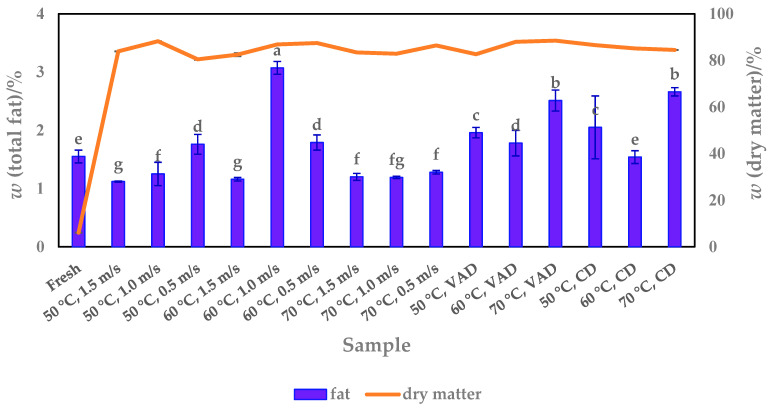
Total fat content versus dry matter content of fresh and dried (HAD, VAD and CD) pumpkin pulp. Values are means ± standard deviations of three (*N* = 3) measurements. Different superscript lowercase letters in the same column indicate significant differences (*p* < 0.05) between drying methods (HAD—hot air drying, VAD—vacuum drying, CD—conductive drying) and conditions.

The results show that pumpkin pulp in both fresh (1.55%) and dried form (1.12–3.07%) contains a low mass percentage of fat (Figure 6) and can therefore be used as a low-fat food source in a balanced diet compared to seeds. These data are comparable to other studies that also confirmed that fresh and dried pumpkin pulp has a low-fat content, ranging from 0.08 to 2.06% [29,30,31,35,36] and 0.04 to 3.60% [36], respectively.

In this study, significantly (*p* < 0.05) different fat values were determined between the dried samples (Figure 6). The low fat content of 1.12 to 1.20% is mainly distributed among the samples dried using HAD at combinations of 50, 60 and 70 °C with an airflow velocity of 1.5 m/s and 70 °C at 1.0 m/s. Slightly higher amounts of fat were distributed at 50 °C (1.76%) and 60 °C (1.79%) at an airflow velocity of 0.5 m/s, while the highest fat value of 3.07% was achieved at 60 °C and 1.0 m/s, so that these parameter combinations can be considered optimal for the production of high-fat dry pulp. As we have written in previous paragraphs, the drying processes and the parameters used are not an isolated process that affects the final chemical composition of the dried food. The chemical composition of food is variable and complex, and this complexity includes properties that change during the drying process. The interactions between water and other components depend on the water content and are therefore responsible for biochemical reactions, physical transformations and mechanical phenomena during drying. In this context, it was mentioned that the influence of water on the final dry matter content is related to the presence of various other compounds with which water can interact. Our results showed that the samples with lower moisture content or higher dry matter content mainly had a higher fat content. Although the results of the statistical analysis showed significant differences between the samples in terms of fat content, the fat values at the same temperature were close to each other in terms of airflow velocities. Samples dried at 50 °C, for example, showed fat values of 1.1 to 1.8% at 1.5, 1.0 and 0.5 m/s, respectively. At 70 °C, the values were 1.2 to 1.3% for 1.5, 1.0 and 0.5 m/s respectively. The slightly larger discrepancy between the airflow velocities occurred at 60 °C, where the values were between 1.2 and 3.1%. We assume that the sample dried at 60 °C and 1.0 m/s had a different structure than that of the other samples, so that a higher fat transfer took place during the extraction, and the higher fat value was achieved due to its better interaction with solvent.

The VAD and CD samples had a higher fat content of 1.78 to 2.51% and 1.54 to 2.66%, respectively, compared to most of the HAD samples. Their distribution in the dried samples is significantly (*p* = 0.023689) influenced by temperature and pressure, such that the highest fat values were obtained at 70 °C, regardless of the pressure used (100 and 1000 mbar). Overall, the data indicate that the use of drying processes depends primarily on the desired amount of fat, so that HAD is preferable for the production of low-fat pumpkin products, for example at an airflow velocity of 1.5 m/s (50, 60 and 70 °C), while VAD, and especially CD at 70 °C, are suitable for high amounts of fat.

### 3.4. Fibre Content

**Figure 7:**
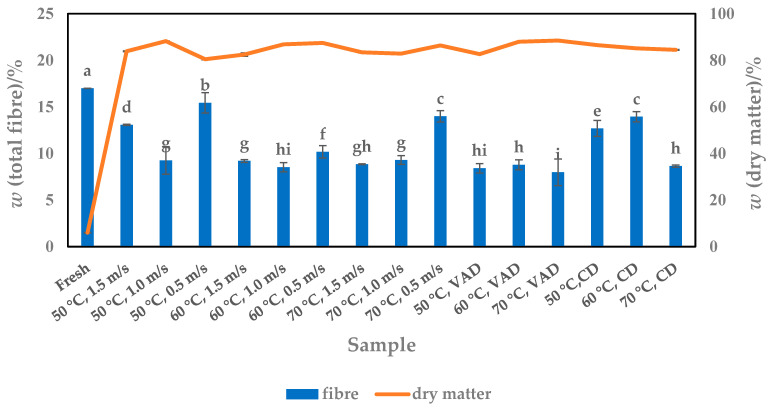
Total fibre content versus dry matter content of fresh and dried (HAD, VAD and CD) pumpkin pulp. Values are means ± standard deviations of three (*N* = 3) measurements. Different superscript lowercase letters in the same column indicate significant differences (*p* < 0.05) between drying methods (HAD—hot air drying, VAD—vacuum drying, CD—conductive drying) and conditions.

Dietary fibre, which consists of soluble and insoluble components and is found in various plants, is an important substance in the daily diet, and its intake has health-promoting effects, such as preventing constipation, reducing body weight and abdominal obesity, chronic inflammation, depression, cardiovascular disease, etc. [46]. To find out whether pumpkin is a valuable source of dietary fibre among other vegetables, its fresh and dried pulp was analyzed. The results show that fresh pulp contains 16.99% dietary fibre, a higher value than dried pulp (7.98–15.43%, db) (Figure 7). It is therefore clear that fresh pulp is the better choice for consumers. However, considering the short shelf life of fresh pulp and the results obtained here with dried pulp, it can be emphasized that the latter could be a promising solution for dietary fibre intake as a dietary supplement [45].

The total dietary fibre content determined in other studies [30,32] is largely comparable to the results of the present study. The values ranged from 8.77% (wb) to 12.24% (db) and 7.85% to 21.95% (db) for fresh [30,33] and dried pulp [30,31], respectively, depending on the *Cucurbita* species and drying conditions. However, the values obtained in this work differ (or not) from those reported by Hussain and co-workers [3], as the amounts detected were not reported as either wb or db. They ranged from 0.42 to 0.56% and 0.81 to 11.25% for fresh and powdered fresh samples, respectively.

As the results show (Figure 6), the fibre content in some of the dried pulp (50 °C, 1.5 m/s and 0.5 m/s, 70 °C, 0.5 m/s and 50 °C, CD and 60 °C, CD) is also high, ranging from 12.69 to 15.43%. Compared to these samples, which could replace the use of fresh pulp, the other dried samples had significantly lower amounts of dietary fibre, ranging from 7.98 to 10.18% (Figure 6). The lower fibre content was mainly distributed among the samples dried at higher temperatures, e.g., 60 and 70 °C and airflow velocities of 1.5 and 1.0 m/s. With some exceptions, increasing both the airflow velocity (from 0.5 to 1.5 m/s) and the drying temperature (from 50 to 70 °C) had a linear and significant (*p* < 0.05) decreasing effect on the total fibre content. According to the post hoc test, temperature had a significantly greater effect on the fibre yield.

In addition to the HAD samples, the pulp processed with VAD and CD had 7.98 to 8.78% and 8.65 to 13.94% total ash, respectively. With *p* = 0.0002, the total fibre content of the dried pulp is dependent on both temperature and pressure. A lower pressure of 100 mbar reduced the fibre content compared to a higher pressure of 1000 mbar, regardless of the applied temperature. In particular, the lowest fibre content at 70 °C was obtained not only for VAD (7.98%) but also for CD pulp (8.65%), demonstrating the significant influence of drying temperature. CD at 50 and 60 °C as well as some of the HAD parameter combinations (especially temperatures of 50 and 70 °C with a lower airflow velocity of 0.5 m/s) would therefore be an option to achieve a fibre content close to that of fresh pulp.

### 3.5. Total Protein Content

Since UAE has been shown to provide higher extraction efficiency of target compounds compared to conventional reflux extraction [47,48], the aim of this study was to use UAE for the extraction of proteins from fresh and dried pumpkin pulp. CE was also used to compare the extraction efficiency of UAE.

The results showed that the total soluble protein content of fresh pulp ranged from 0.87 to 2.30% (wb), depending on the extraction time in CE (20 and 40 min) and UAE (10, 20 and 40 min), which corresponded to a total protein content of 14.70 to 38.20% (db) (Table 3). Since protein extraction from fresh and dried pumpkin pulp was performed using UAE and CE, for which there are no scientific reports, the protein extraction efficiency presented here was compared with the results obtained using other methods, such as the Kjeldahl method, which is commonly used for protein determination in pumpkin samples. As reported by Hussain et al. [3] in a review paper, the protein content varies between fresh (0.29–1.13%) and dried (1.30–15.50%) pulp, so the results obtained in this study were within these limits. In addition to this review, other authors (Table 2) have reported values of 0.49 to 1.6% (wb) and 12.02% (wb) and 10.88 to 17.30% (db) for fresh [17,20,23,25] and dried pulp [30,32,35], respectively, confirming the efficiency of the drying and extraction methods presented here.

In this work, with the exception of sample UAE, 40 min, fresh pulp was found to contain a lower mass fraction of proteins (14.70–19.44%) compared to most dried samples (max. value is 22.34%), regardless of the drying methods and parameters and extraction times used (Table 3). The differences between protein content were significant (*p* < 0.05) due to the different temperatures (50, 60 and 70 °C) and airflow velocities (0.5, 1.0 and 1.5 m/s) at HAD and temperatures of 50, 60 and 70 °C at constant pressure at VAD (100 mbar) and CD (1000 mbar).

**Figure 8:**
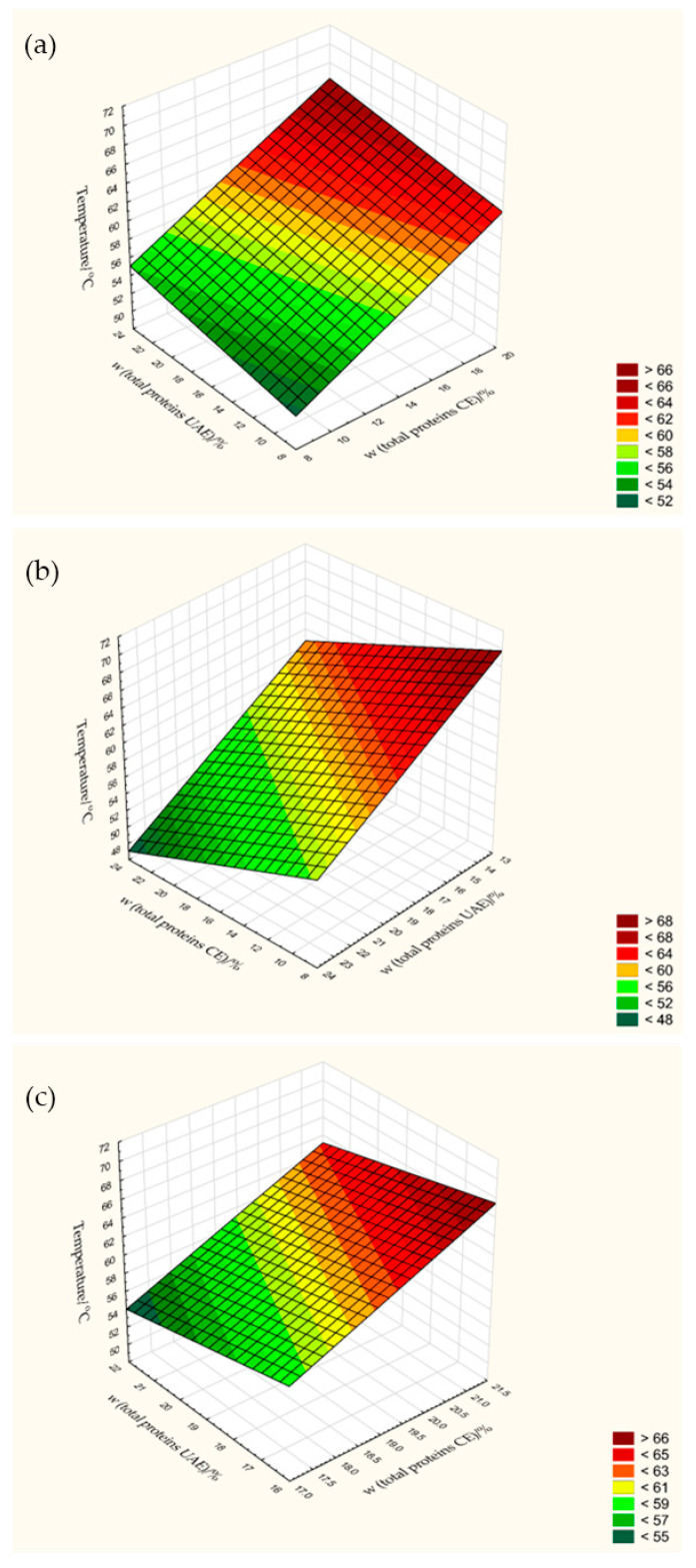
Influence of temperature (50, 60 and 70 °C) on the total protein content of pumpkin pulp according to HAD (**a**), VAD (**b**) and CD (**c**).

ANOVA analysis revealed that the increase in temperature had no significant effect (*p* = 0.14426) on protein content, regardless of airflow velocity and extraction time at CE, although the maximum protein yield was obtained at a temperature of 70 °C (Figure 8a) for 40 min of extraction (Figure S1a, Supplementary Materials). In contrast to the HAD samples subjected to CE, a positive correlation between drying temperature and protein content was observed in the HAD samples subjected to UAE. Temperature significantly (*p* = 0.00193) increased the total protein yield up to 15.51% at 70 °C regardless of the extraction time. In addition to temperature, airflow velocity also increased the protein yield, and as shown by 3D scattering, its influence is more pronounced for pulp subjected to UAE (Figure S2, Supplementary Materials) than for CE. In addition to temperature and airflow velocity, the effect of extraction time in UAE on protein content is also significant (*p* = 0.02272), which means that further increasing the time up to 20 min (Figure S1a, Supplementary Materials) decreases the amount of protein, which is probably due to the cavitation effect of ultrasound. As the extraction time increased, the number of cavitation bubbles and the total cavitation yield in the solvent increased, and their excessive force destroyed the pulp cells, leading to protein degradation [49]. Therefore, it is likely that a temperature of 70 °C for HAD pulp in combination with an extraction time of 20 min in UAE would be an optimal processing parameter to achieve a high protein yield.

A high protein content (19.93–22.35%) is also achieved with VAD at 50 °C, so that this drying method could be used for further protein intensification depending on the extraction method (CE and UE) and time (Figure 8b). A high protein content at 50 °C is also achieved with CD, which is between 18.48 and 21.22% depending on the extraction method and time. Compared to VAD, where temperature increases significantly influenced protein degradation in samples dried at 60 and 70 °C, CD under the constant pressure of 1000 mbar can keep the protein content constant despite the temperature increase (Figure 8c). However, both drying methods at a temperature between 50 and 60 °C maximized the protein yield, independent of CE and UAE.

Overall, we assume that the differences in protein content of finished pumpkin samples may lie in the differences in structure after applying the three different drying methods. As a result of drying, the obtained samples may contain voids, cracks and pores with different diameters. Therefore, the capacity of solvent to extract the protein may not be the same for each dried sample, i.e., the mass transfer of protein to solvent is different, resulting in different protein values.

In addition to the drying temperature, the extraction time was also shown to have a significant influence on the protein yield of VAD and CD pulp. The differences between 20 and 40 min for CE were less pronounced in the case of VAD pulp, so that both times gave a good result (Figure S1b, Supplementary Materials). Regardless of the extraction times used in UAE (10, 20 and 40 min), VAD pulp showed that prolonged extraction up to 40 min resulted in a significantly higher (*p* = 0.0199) protein yield. An extraction time of longer than 40 min was not used in this work, as ultrasound-induced cavitation can denature proteins, leading to chemical and physical changes [50].

Although for VAD pulp extracted for 20 and 40 min, the differences between protein content were minimal and for CD pulp this means that 40 min is more efficient (Figure S1c, Supplementary Materials). Thus, the further choice of parameters for protein extraction using these two drying methods under pressure would be a temperature between 50 and 60 °C for 40 min using UAE.

### 3.6. Total Sugar Content

The total sugars in fresh and dried pumpkin pulp were also extracted using UAE and CE. The results show (Table 4) that fresh pumpkin pulp contains 2.61 to 5.67% (wb) sugar, depending on the extraction method and time. Compared to fresh pulp (43.30–94.13%, db), dried pulp contains 21.12 to 79.22% (db) total sugars, depending on the drying processes and conditions, extraction method and time. With the exception of the sugar value of 94.13% in the fresh pulp (sample UAE, 40 min), the sugar content in most of the dried samples extracted using CE for 20 and 40 min and UAE for 10 and 20 min was higher (48.77–79.22%, db) than in the fresh pulp (43.30–61.359%, db) (Table 4), suggesting that dried pulp could be used as an excellent source of added sugar, for example, to improve the sweet taste of various foods and reduce the need for added sugar. This could be of great importance for diabetics, as pumpkin is considered a hypoglycaemic plant to control blood glucose levels [51].

**Figure 9:**
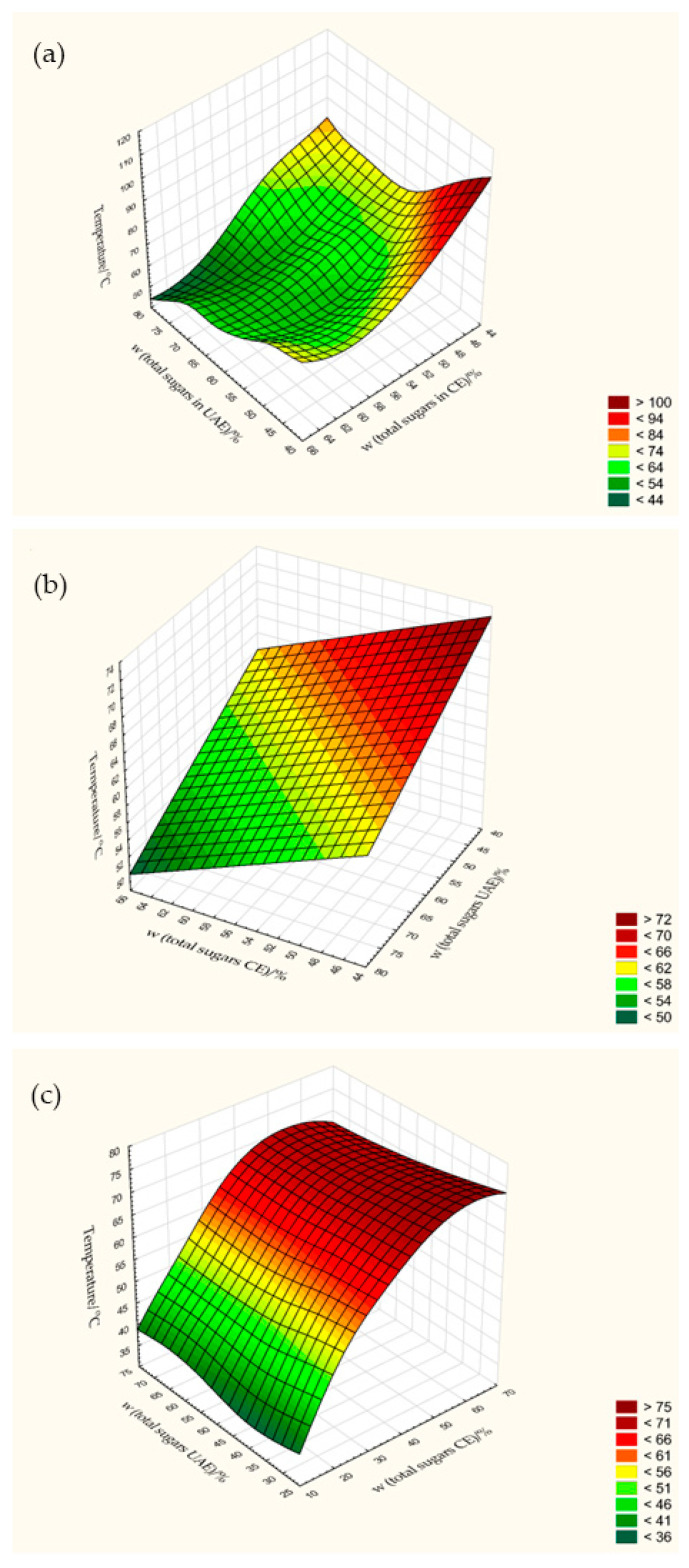
Influence of temperature (50, 60 and 70 °C) on the total sugar content of pumpkin pulp according to HAD (**a**), VAD (**b**) and CD (**c**).

As mentioned in the analysis of proteins, there are also no reports on the use of CE and UAE for sugar extraction. However, regardless of the methodology used, the results obtained in this work are comparable to total sugars determined in other works. Armesto et al. [35] found 2.15 to 2.90% total sugars in fresh pulp, depending on the *Cucurbita* variety and cultivation method. Guine et al. [30] and Montesano et al. [29] reported values of 4.7% and 4.9% (wb) for *Cucurbita maxima* spp. For dried pumpkin pulp, Sojak et al. [27] also reported high total sugar content ranging from 31.20 to 89.52% (db), depending on the type of drying (chamber, tunnel and fluidized bed dryers) and the temperatures applied (40, 50, 60, 70 and 80 °C). On the other hand, Guine et al. [30] obtained lower values of 17.09 to 18.66% (db) depending on the drying conditions compared to the results of the present study. The lowest sugar content (29.03 to 38.73%) of HAD pulp was obtained at 50 °C, 1.5 m/s and 70 °C, 0.5 m/s by CE (20 and 40 min) and UAE (10 and 20 min). In contrast to these samples, the highest mass fractions of total sugars were obtained by combining temperatures of 70 °C with air flow velocities of 1.0 and 1.5 m/s depending on the extraction time used in CE. At extraction times of 20 and 40 min, values of 70.11 and 79.22% and 68.53 and 70.86% were obtained at velocities of 1.0 and 1.5 m/s, respectively. This increasing effect on sugar content with increasing airflow velocity is also confirmed by Figure S3 (Supplementary Materials) for pulp extracted using both UAE and CE. In addition to airflow velocity, increasing the extraction time to 40 min also led to a positive correlation with the sugar content, with the highest values being achieved at 40 min regardless of the drying temperature (Figure S4a, Supplementary Materials). However, regardless of the extraction time and airflow velocity, the 3D scatter plot (Figure 9a) showed that the total sugar content of the HAD samples extracted using CE decreased with increasing drying temperature. The effect of temperature on total sugar content is also significant (*p* = 0.00016) for HAD pulp treated with UAE. The highest increases in sugar yield were obtained for samples dried between 60 and 70 °C, reaching a maximum value of about 58.5% at 70 °C. In addition to the temperature, increasing the extraction time from 10 to 40 min when using UAE also led to a significant increase (*p* = 0.00003) in the sugar content from around 48.2 to 58.6%. Thus, a higher sugar yield can be achieved using UAE after reaching the temperature of 70 °C for 40 min compared to CE.

In addition to HAD, a high sugar content of 49.24 to 67.54% (db) was also achieved with VAD pulp, depending on the drying temperature, the method and the time for sugar extraction. It can be seen (Figure S4b, Supplementary Materials) that increasing the extraction time from 20 to 40 min in CE led to a change in the sugar amounts (*p* = 0.00002), so that a higher sugar content was obtained with a longer extraction time of 40 min. However, when the temperature was increased during drying, a sharp drop in sugar content was observed, corresponding to an increase in temperature from 50 to 70 °C (Figure 9b). In the case of VAD pulp subjected to UAE, the results of the factorial ANOVA performed show that extraction time and drying temperature together had a significant effect on sugar yield (*p* = 0.0002). A minimum extraction time of 10 min did not change the sugar content, regardless of the temperature used. A further increase in extraction time to 20 min led to an increase in sugar content, which reached its maximum at a temperature of 70 °C (67.54%). The combination of the maximum temperature of 70 °C and the longest extraction time led to a significant decrease in sugar content (51.57%), possibly due to the cavitation effect of the ultrasound.

CD also gave good results (49.38–62.41%, depending on the extraction method) at a temperature of 70 °C, while the temperatures of 50 and 60 °C were not suitable due to the lower sugar yield, especially when CE was used (21.12–28.79%). The higher sugar values were obtained using UAE (34.58–62.41%), especially for samples extracted for 40 min. As confirmed by the 3D scatter plot (Figure S4c, Supplementary Materials), extraction time in CE had no significant effect on total sugar content, but a stronger correlation (*p* = 0.00001) was observed between temperature and sugar levels (Figure 9c), with a maximum value of 55.98% at a drying temperature of 70 °C for 40 min of extraction. A similar conclusion can be drawn for CD pulp subjected to UAE, i.e., a maximum drying temperature of 70 °C led to maximum sugar amounts, regardless of the extraction time. A longer extraction time led to an increase in sugar content, but there were no statistical differences between extraction times of 20 and 40 min for the sample dried at maximum temperature, so the optimum parameters for drying and extraction would be 70 °C and 20 min, respectively.

Overall, the results showed that a temperature of 70 °C is suitable for further drying of pumpkin pulp using HAD and CD in combination with a 40 min extraction time using UAE for VAD and CD pumpkin pulp. However, the temperature of 50 °C and a 40 min extraction time using UAE would be the more suitable option for HAD pulp.

### 3.7. Texture Profile

It is known that the evaporation of water during drying increases the concentration of solids and changes the mechanical properties of the materials to an extent that depends on the drying conditions. To understand texture from a sensory point of view, it is necessary to know the changes in mechanical properties, as they are closely linked to the textural and sensory properties of the food. Numerous researchers have studied the mechanical properties of fresh and dried pumpkin and other vegetables during convective drying. In broad terms, their results consistently indicate a transition from a texture with low hardness due to the water content to a more rigid state after drying. It is also observed that the structure of the cell matrix changes from a plastic behaviour to a more elastic or crunchy behaviour [52].

Table 5 shows the effects of HAD, VAD and CD methods and conditions on the hardness, elasticity and working performance of fresh and dried pumpkin pulp. VAD is known to maintain the hardness of dried products. The reduced pressure in the vacuum chamber allows for faster moisture removal at lower temperatures and minimizes the structural changes that can lead to softening. In contrast to VAD, the hardness of pumpkin dried with CD and HAD depends significantly on the much higher drying temperatures, which can lead to structural changes in the dried material. All drying methods significantly (*p* < 0.05) reduced the hardness, elasticity and work values compared to fresh pumpkin pulp. Similar results for HAD are presented in the study of Monteiro et al. [53], where dried pumpkin samples showed a compact and rigid structure that broke at about 60% of maximum elongation. The highest hardness value of 27.24 N was obtained for fresh pulp compared to the dried samples (11.29–18.72 N), indicating that the dried samples were more porous and had a softer texture than the fresh ones, whose structure was hard and more fibrous. The pumpkin pulp dried using HAD had the lowest hardness values (11.29–14.29 N), indicating that it is more sensitive to HAD than to VAD and CD, which had values of 14.90 to 17.24 N and 15.48 to 18.72 N, respectively, depending on the drying parameters. This is consistent with studies by Guiné and Barroca [52], which showed a decrease in hardness after drying pumpkin pulp.

Similar to the hardness value, the elasticity of the fresh pulp was also high (3.47 mm) compared to the majority of the dried samples (1.21–3.07 mm). Slightly higher values (3.07, 3.20 and 3.99 mm) near the fresh pulp were exhibited by the HAD samples dried at higher temperatures (60 and 70 °C) and airflow velocities of 1.0 and 1.5 m/s. The work as a measure of the energy required to break the strength of the internal bonds of the sample was also higher for fresh pulp (46.77 mJ) than for dried pulp (9.45 to 15.55 mJ). The samples dried at 50 °C, including HAD, VAD and CD, had lower work values (9.45–9.78 mJ) than the other dried pulp (11.11–15.55 mJ). Thus, in contrast to elasticity, the samples dried at the lower temperature of 50 °C had lower work done values, suggesting a significant temperature effect. It is evident that the lowest drying temperatures had the least effect on textural properties compared to fresh pumpkin. This is also confirmed in the work of Henriques et al. [54], in which all drying treatments tested caused a significant decrease in hardness, with the least changes occurring at 40 °C. Although vacuum drying is known to preserve the texture of fruits and vegetables, VAD has a higher degree of hardness and lower elasticity compared to CD, which is also confirmed by the lower work needed for mastication. Depending on the drying temperature and duration, CD generally leads to greater changes in texture due to the degradation of the cell structure at high temperatures, which should result in a softer and more brittle texture. The greatest change in hardness was observed at the maximum drying temperature and air flows of 1.0 and 1.5 m/s. At the same parameters, the elasticity remains excellent, which is almost the same as for the fresh samples. This is to be expected since VAD usually results in products that are more resistant and elastic due to the preservation of their integrity and the lower probability of collapse or shrinkage. Based on the plans for further processing of dried pumpkin, optimum parameters were achieved with a temperature of 70 °C and an airflow of 0.5 m/s, which provide an excellent intermediate product for the further production of pumpkin powder in mills.

## 4. Conclusions

This work shows that it is possible to extend the shelf life of fresh pumpkin while maintaining good quality of the dried pulp by using HAD, VAD and CD processes. However, which drying methods and which combination of drying parameters are preferable depends primarily on the specific composition(s) and further use of the pumpkin.

In this context, a temperature between 50 and 60 °C and an airflow velocity of 1.0 m/s would be the optimal parameters for the HAD process to reduce moisture and obtain a high ash and fat content. A good source of fibre is pulp obtained using HAD and CD processes at 50 °C and an airflow velocity of 0.5 m/s and at a temperature of 50 and 60 °C, respectively. In contrast to the mild conditions suitable for obtaining the fibre content of near-fresh pulp, the high temperature of 70 °C for VAD pulp gave the lowest moisture content with low ash and fibre content and high fat content. HAD pulp at 70 °C and VAD and CD pulp processed between 50 and 60 °C resulted in a high protein content. The highest temperature of 70 °C used for HAD and CD pulp is also optimal for achieving a high sugar content, while a temperature of 50 °C is best for VAD pulp. In addition to drying, UAE has also proven to be a suitable method for determining protein and sugar content as a function of extraction time. For protein extraction of HAD pulp, 20 min would be optimal, while for pulp dried with VAD and CD, 40 min is the better choice. For sugar extraction, 40 min is optimal for all three dried pulp types.

In addition to the chemical profiles, drying led to strong changes in the textural properties of the pumpkin pulp, resulting in lower values for hardness, elasticity and work compared to fresh pulp.

## Figures and Tables

**Table 1:** Experimental parameters used in hot air drying (HAD, vacuum drying (VAD) and conductive drying (CD).

Drying Method	Process Parameters			Sample Code
	Temperature	Airflow Velocity	Pressure	
	(°C)	(m/s)	(mbar)	
HAD	50	1.5	1000	50 °C, 1.5 m/s
		1.0		50 °C, 1.0 m/s
		0.5		50 °C, 0.5 m/s
	60	1.5		60 °C, 1.5 m/s
		1.0		60 °C, 1.0 m/s
		0.5		60 °C, 0.5 m/s
	70	1.5		70 °C, 1.5 m/s
		1.0		70 °C, 1.0 m/s
		0.5		70 °C, 0.5 m/s
VAD	50	0.0	100	50 °C, VAD
	60			60 °C, VAD
	70			70 °C, VAD
CD	50	0.0	1000	50 °C, CD
	60			60 °C, CD
	70			70 °C, CD

**Table 2:** Summary of previous fresh and dried experiments conducted on pumpkins.

Pulp	Moisture (%)	Ash (%)	Fat (%)	Fibre (%)	Protein (%)	Sugars (%)	Reference
*C. maxima*							
Fresh	82.10–84.09 (wb)	-	-	-	-	-	[13]
	-	-	-	-	0.49–0.76 (wb)	8.9–10.7 (wb)	[24]
	19.66 (wb)					11.62 (wb)	[25]
	85.33 *	3.21 *	-	-	1.19 *	-	[26]
	82.50% (wb)	0.84 (wb)	0.08 (wb)	-	1.28 (wb)	4.90 (wb)	[27]
	91.9 (wb)	1.1 (wb)	0.2 (wb)	1.0 (wb)	1.6 (wb)	4.7 (wb)	[28]
	-	13.99 (db)	2.04 (db)	12.24 (db)	17.3 (db)	52.84 (db)	
Dried	5.47–10.21 (db)	-	-	-	-	-	[13]
	91.15–93.45 (wb)					31.20–89.52 (db)	[25]
	7.62 *	6.25 *	-	-	1.81*	-	[26]
	2.7–4.6 (wb)	17.75–18.62 (db)	1.24–2.06 (db)	7.85–9.69 (db)	15.83–16.68 (db)	17.09–18.66 (db)	[28]
	9.16–9.25 *	0.36–0.55 *	0.14–0.19 *	0.16–0.35 *	1.05–1.06 *	0.35–1.05 *	[29]
	7.10–8.31 (wb)	8.06–8.14 (wb)	2.17–2.19 (wb)	25.12–25.45 (wb)	8.53–8.64 (wb)	48.29–48.83 (wb)	[30]
*C. moshata*							
Fresh	87.78 (wb)	6.16 (wb)		8.77 (wb)	12.02 (wb)	-	[31]
	92.34 (wb)						[32]
	88.90–91.58 *	1.03–1.31 *	0.12–0.17 *	2.20–2.97 *	0.12–0.17 *	2.15–2.90 *	[33]
Dried	9.69 (db)	5.91 (db)		9.03 (db)	10.88 (db)	-	[31]
	10.81 (wb)						[32]
	7.06 (wb)	6.37 (db)	1.88 (db)	21.95 (db)	11.08 (db)	51.88 (db)	[34]

wb—wet base, db—dry base, *—neither specified as wb nor db.

**Table 3:** Total protein content in fresh and dried (HAD, VAD and CD) pumpkin pulp obtained after conventional reflux extraction (CE) and ultrasound-assisted extraction (UAE).

Sample Code	*w* (Total Protein)/%									
	CE				UAE					
	*t* (Extraction)/min									
	20		40		10		20		40	
Fresh	15.22 ± 1.27 ^cD^	**(0.92)**	14.70 ± 0.02 ^dDE^	**(0.89)**	17.09 ± 0.95 ^cdC^	**(1.03)**	19.44 ± 2.34 ^bBC^	**(1.17)**	38.20 ± 5.05 ^aA^	**(2.30)**
50 °C, 1.5 m/s	20.34 ± 2.13 ^aA^	**(17.06)**	20.57 ± 0.58 ^aA^	**(17.25)**	11.67 ± 1.05 ^hD^	**(9.79)**	16.44 ± 0.33 ^eB^	**(13.79)**	12.54 ± 1.41 ^ijC^	**(10.51)**
50 °C, 1.0 m/s	13.49 ± 0.65 ^cD^	**(11.90)**	13.19 ± 0.65 ^efDE^	**(11.64)**	15.39 ± 0.57 ^eB^	**(13.58)**	16.99 ± 0.49 ^deA^	**(14.99)**	15.23 ± 0.53 ^hBC^	**(13.44)**
50 °C, 0.5 m/s	15.95 ± 1.38 ^bA^	**(12.83)**	14.17 ± 0.34 ^eC^	**(11.40)**	14.34 ± 0.95 ^eB^	**(11.53)**	11.09 ± 0.44 ^iD^	**(8.92)**	9.72 ± 0.16 ^lE^	**(7.80)**
60 °C, 1.5 m/s	15.23 ± 0.17 ^cE^	**(12.56)**	16.73 ± 0.19 ^cD^	**(13.80)**	18.41 ± 0.17 ^bB^	**(15.18)**	17.30 ± 0.19 ^cdC^	**(14.27)**	19.23 ± 0.18 ^dA^	**(15.86)**
60 °C, 1.0 m/s	11.73 ± 0.96 ^deC^	**(10.19)**	11.43 ± 0.93 ^gCD^	**(9.93)**	10.85 ± 0.59 ^hD^	**(9.42)**	15.44 ± 0.39 ^fA^	**(13.41)**	14.21 ± 0.41 ^hiB^	**(12.34)**
60 °C, 0.5 m/s	12.94 ± 0.56 ^cdA^	**(11.32)**	13.07 ± 0.88 ^fAB^	**(11.43)**	12.48 ± 0.15 ^ghB^	**(10.92)**	14.51 ± 1.91 ^ghA^	**(12.69)**	11.09 ± 0.39 ^kC^	**(9.69)**
70 °C, 1.5 m/s	15.60 ± 0.84 ^bcE^	**(13.01)**	16.47 ± 0.16 ^cdD^	**(13.74)**	18.25 ± 0.18 ^bB^	**(15.22)**	17.59 ± 0.53 ^cdC^	**(14.53)**	18.56 ± 0.18 ^eA^	**(15.48)**
70 °C, 1.0 m/s	14.81 ± 1.21 ^cD^	**(12.27)**	16.88 ± 0.16 ^cBC^	**(13.99)**	18.40 ± 0.18 ^bA^	**(15.25)**	16.56 ± 0.35 ^deC^	**(13.72)**	17.27 ± 0.36 ^fB^	**(14.31)**
70 °C, 0.5 m/s	17.53 ± 0.39 ^bB^	**(15.14)**	20.05 ± 0.49 ^aA^	**(17.28)**	14.32 ± 0.16 ^eCD^	**(12.37)**	14.00 ± 0.33 ^hD^	**(12.09)**	11.92 ± 0.64 ^jE^	**(10.30)**
50 °C, VAD	19.94 ± 0.72 ^aD^	**(16.47)**	19.93 ± 0.97 ^aD^	**(16.46)**	21.22 ± 1.40 ^aBC^	**(17.54)**	22.03 ± 0.43 ^aB^	**(18.20)**	22.35 ± 0.37 ^bA^	**(18.47)**
60 °C, VAD	11.63 ± 1.11 ^eCD^	**(10.23)**	10.88 ± 0.78 ^gC^	**(** **9.57)**	12.91 ± 0.53 ^gB^	**(11.35)**	14.77 ± 0.36 ^gA^	**(12.99)**	14.85 ± 0.46 ^hA^	**(13.06)**
70 °C, VAD	12.75 ± 0.55 ^dD^	**(** **11.28)**	13.30 ± 0.80 ^eC^	**(** **11.76)**	13.94 ± 0.46 ^fBC^	**(12.33)**	16.42 ± 0.46 ^eA^	**(14.53)**	16.33 ± 0.46 ^gA^	**(14.45)**
50 °C, CD	19.35 ± 1.40 ^aB^	**(16.74)**	18.85 ± 0.46 ^bC^	**(16.30)**	18.48 ± 0.44 ^bC^	**(15.99)**	19.25 ± 0.59 ^bB^	**(16.65)**	21.22 ± 0.60 ^cA^	**(18.35)**
60 °C, CD	18.16 ± 0.81 ^abC^	**(15.46)**	19.12 ± 0.28 ^bA^	**(16.28)**	16.78 ± 0.53 ^dD^	**(14.29)**	17.78 ± 0.70 ^cCD^	**(15.14)**	18.72 ± 0.38 ^eB^	**(15.94)**
70 °C, CD	19.32 ± 0.65 ^aB^	**(16.32)**	19.69 ± 0.82 ^abAB^	**(16.63)**	17.68 ± 0.46 ^bcC^	**(14.93)**	19.79 ± 0.73 ^bAB^	**(16.72)**	20.39 ± 1.06 ^cdA^	**(17.23)**

Values are mean ± standard deviations of three (*N* = 3) measurements expressed on a dry basis. The bolded numbers in brackets correspond to a mean value of examined samples, before conversion on dry basis. Different superscript lowercase letters within rows are significantly different (*p* < 0.05). Different superscript uppercase letters within columns are significantly different (*p* < 0.05).

**Table 4:** Total sugar content in fresh and dried (HAD, VAD and CD) pumpkin pulp obtained after conventional reflux extraction (CE) and ultrasound-assisted extraction (UAE).

Sample Code	*w* (Total Sugar)/%									
	CE				UAE					
	*t* (Extraction)/min									
	20		40		10		20		40	
Fresh	43.30 ± 9.95 ^eD^	**(2.73)**	44.04 ± 4.21 ^eD^	**(2.61)**	49.51 ± 8.47 ^cC^	**(2.96)**	61.35 ± 4.02 ^bB^	**(3.70)**	94.13 ± 6.66 ^aA^	**(5.67)**
50 °C, 1.5 m/s	35.23 ± 5.29 ^fgD^	**(29.54)**	38.11 ± 4.80 ^fBC^	**(31.97)**	35.84 ± 1.95 ^eD^	**(30.06)**	38.83 ± 0.79 ^hB^	**(32.57)**	45.31 ± 2.41 ^hA^	**(38.00)**
50 °C, 1.0 m/s	45.74 ± 5.62 ^eE^	**(40.35)**	52.57 ± 6.23 ^cdD^	**(46.38)**	52.97 ± 4.38 ^cCD^	**(46.73)**	53.75 ± 3.56 ^dB^	**(47.42)**	57.35 ± 4.18 ^fA^	**(50.59)**
50 °C, 0.5 m/s	52.03 ± 6.92 ^cC^	**(41.86)**	66.07 ± 14.21 ^aA^	**(53.15)**	50.02 ± 3.90 ^cD^	**(40.24)**	50.99 ± 4.01 ^eD^	**(41.02)**	61.39 ± 3.74 ^eB^	**(49.39)**
60 °C, 1.5 m/s	62.16 ± 3.35 ^bB^	**(51.26)**	65.46 ± 5.40 ^abA^	**(53.99)**	62.42 ± 2.47 ^bB^	**(51.48)**	64.11 ± 7.18 ^abA^	**(52.87)**	62.30 ± 2.67 ^eB^	**(51.38)**
60 °C, 1.0 m/s	40.15 ± 6.14 ^efC^	**(34.87)**	56.13 ± 6.17 ^bA^	**(48.74)**	48.62 ± 3.56 ^cB^	**(42.22)**	41.46 ± 2.94 ^gC^	**(36.00)**	52.05 ± 6.85 ^gAB^	**(45.20)**
60 °C, 0.5 m/s	59.59 ± 7.25 ^bA^	**(52.12)**	48.77 ± 5.65 ^dB^	**(42.65)**	40.72 ± 3.34 ^eC^	**(35.61)**	46.21 ± 1.56 ^fB^	**(40.41)**	56.83 ± 4.59 ^fA^	**(49.70)**
70 °C, 1.5 m/s	79.22 ± 2.03 ^aB^	**(66.07)**	70.86 ± 3.22 ^aD^	**(59.10)**	75.30 ± 4.82 ^aC^	**(62.80)**	63.05 ± 6.52 ^abE^	**(52.58)**	83.23 ± 4.58 ^bA^	**(69.42)**
70 °C, 1.0 m/s	70.11 ± 5.55 ^bA^	**(58.10)**	68.53 ± 3.61 ^aA^	**(56.78)**	70.69 ± 8.84 ^aA^	**(58.57)**	70.29 ± 3.43 ^aA^	**(58.25)**	69.98 ± 2.26 ^cdA^	**(57.98)**
70 °C, 0.5 m/s	38.73 ± 8.79 ^fB^	**(33.45)**	34.07 ± 10.89 ^fBC^	**(29.43)**	29.03 ± 2.76 ^eC^	**(25.08)**	52.55 ± 7.63 ^deA^	**(45.39)**	57.24 ± 6.32 ^fA^	**(49.44)**
50 °C, VAD	58.86 ± 5.83 ^bB^	**(48.64)**	58.41 ± 3.47 ^bcB^	**(48.27)**	61.39 ± 4.34 ^bB^	**(50.73)**	58.68 ± 3.36 ^cB^	**(48.49)**	65.79 ± 8.02 ^dA^	**(54.36)**
60 °C, VAD	57.84 ± 6.60 ^bcB^	**(50.87)**	60.89 ± 3.53 ^bcA^	**(53.55)**	61.02 ± 4.96 ^bcA^	**(53.67)**	61.25 ± 3.23 ^bA^	**(53.87)**	61.02 ± 5.63 ^efA^	**(53.67)**
70 °C, VAD	49.24 ± 3.13 ^eD^	**(43.57)**	60.95 ± 6.18 ^bB^	**(53.93)**	60.26 ± 5.39 ^bcC^	**(53.32)**	67.54 ± 6.34 ^abA^	**(59.76)**	51.57 ± 6.54 ^gD^	**(45.63)**
50 °C, CD	23.41 ± 2.44 ^hD^	**(20.25)**	21.12 ± 0.91 ^fD^	**(18.27)**	45.77 ± 1.22 ^dC^	**(39.60)**	47.84 ± 2.84 ^fB^	**(41.39)**	61.19 ± 5.42 ^efA^	**(52.93)**
60 °C, CD	28.72 ± 0.54 ^hD^	**(24.45)**	28.79 ± 1.77 ^fD^	**(24.51)**	35.70 ± 3.08 ^eBC^	**(30.39)**	34.58 ± 4.24 ^hC^	**(29.44)**	44.17 ± 4.08 ^hA^	**(37.60)**
70 °C, CD	49.86 ± 1.89 ^deC^	**(42.12)**	55.98 ± 7.61 ^cBC^	**(47.29)**	49.39 ± 1.14 ^cC^	**(41.72)**	62.41 ± 6.96 ^bA^	**(52.72)**	57.38 ± 0.87 ^fB^	**(48.46)**

Values are mean ± standard deviations of three (*N* = 3) measurements expressed on a dry basis. The bolded numbers in brackets correspond to a mean value of examined samples, before conversion on dry basis. Different superscript lowercase letters within rows are significantly different (*p* < 0.05). Different superscript uppercase letters within columns are significantly different (*p* < 0.05).

**Table 5:** Textural parameters of fresh and dried (HAD, VAD and CD) pumpkin pulp.

Sample Code	Hardness/(N)	Elasticity/(mm)	Work/(mJ)
Fresh	27.74 ± 4.12 ^a^	3.47 ± 0.26 ^a^	46.77 ± 6.11 ^a^
50 °C, 1.5 m/s	12.18 ± 0.64 ^f^	1.90 ± 0.10 ^g^	9.45 ± 1.08 ^e^
50 °C, 1.0 m/s	12.21 ± 0.87 ^f^	1.93 ± 0.10 ^f^	9.52 ± 1.12 ^e^
50 °C, 0.5 m/s	13.16 ± 1.25 ^ef^	1.96 ± 0.07 ^f^	9.76 ± 1.75 ^e^
60 °C, 1.5 m/s	12.35 ± 2.11 ^f^	3.20 ± 0.57 ^ab^	11.80 ± 3.33 ^c^
60 °C, 1.0 m/s	13.28 ± 1.17 ^e^	2.10 ± 0.09 ^e^	11.11 ± 0.61 ^e^
60 °C, 0.5 m/s	13.37 ± 2.41 ^de^	2.14 ± 0.12 ^e^	11.43 ± 0.86 ^de^
70 °C, 1.5 m/s	11.48 ± 2.50 ^f^	3.99 ± 1.29 ^a^	10.95 ± 2.06 ^e^
70 °C, 1.0 m/s	11.29 ± 4.92 ^g^	3.07 ± 0.86 ^bc^	8.54 ± 2.84 ^f^
70 °C, 0.5 m/s	14.29 ± 1.83 ^d^	1.62 ± 0.19 ^i^	12.02 ± 1.19 ^d^
50 °C, VAD	14.90 ± 2.97 ^d^	1.81 ± 0.33 ^h^	9.77 ± 0.87 ^e^
60 °C, VAD	17.24 ± 1.52 ^b^	2.68 ± 0.60 ^cd^	14.98 ± 0.73 ^c^
70 °C, VAD	15.97 ± 2.09 ^c^	1.75 ± 0.52 ^h^	11.57 ± 2.59 ^d^
50 °C, CD	15.48 ± 4.42 ^cd^	2.49 ± 0.16 ^d^	9.78 ± 4.22 ^e^
60 °C, CD	16.31 ± 0.66 ^c^	1.21 ± 0.42 ^j^	13.43 ± 0.86 ^c^
70 °C, CD	18.72 ± 1.29 ^b^	1.38 ± 0.45 ^j^	15.55 ± 0.36 ^b^

Different superscript lowercase letters within rows are significantly different (*p* < 0.05).

## Data Availability

Data is contained within the article or supplementary material.

## Supplementary Materials

The following supporting information can be downloaded at: https://www.mdpi.com/article/10.3390/foods13040520/s1, Figure S1: Influence of extraction time in UAE (10, 20 and 40 min) and CE (20 and 40 min) on the total protein content of pumpkin pulp processed using HAD (a), VAD (b) and CD (c); Figure S2: Influence of airflow velocity used in HAD (0.5, 1.0 and 1.5 m/s) on the total protein content of pumpkin pulp subjected to UAE and CE; Figure S3: Influence of airflow velocity used in HAD (0.5, 1.0 and 1.5 m/s) on the total sugar content of pumpkin pulp subjected to UAE and CE. Figure S4: Influence of extraction time in UAE (10, 20 and 40 min) and CE (20 and 40 min) on the total sugar content of pumpkin pulp processed using HAD (a), VAD (b) and CD (c).

## Abbreviations

HAD: hot air drying; VAD: vacuum drying; CD: conductive drying; UAE: ultrasound-assisted extraction; CE: conventional extraction
