# Supplementary material for: Influence of Different Drying Processes on the Chemical and Texture Profile of Cucurbita maxima Pulp
Source: Foods. 2024 Feb 8;13(4):520. doi: 10.3390/foods13040520 (PMC10888355; doi:10.3390/foods13040520)
Supplement: Supplementary file 1 [file foods-13-00520-s001.zip › foods-2825498-supplementary.pdf]

foods

Article

Influence of Different Drying Processes on the Chemical and Texture Profile of *Cucurbita maxima* Pulp

Antonela Ninčević Grassino, Sven Karlović, Lea Šošo, Filip Dujmić, Marija Badanjak Sabolović, Marko Marelja, Mladen Brnčić*

University of Zagreb Faculty of Food Technology and Biotechnology, Pierottijeva 6, 10 000 Zagreb; aninc@pbf.hr (A.N.G.); skarlovi@pbf.hr (S.K.); leasoso96@gmail.com; filip.dujmic@pbf.unizg.hr (F.D.); mbadanjak@pbf.hr (M.B.S.); mmarelja@pbf.hr (M.M.) mbrncic@pbf.hr (M.B.)

* Correspondence: mbrncic@pbf.hr (M.B.)

Table of Contents

Figure S1. Influence of extraction time in UAE (10, 20 and 40 min) and CE (20 and 40 min) on the total protein content of pumpkin pulp processed with HAD **(a)**, VAD **(b)** and CD **(c)**.

Figure S2. Influence of airflow velocity used in HAD (0.5, 1.0 and 1.5 m/s) on the total protein content of pumpkin pulp subjected to UAE and CE.

Figure S3. Influence of extraction time in UAE (10, 20 and 40 min) and CE (20 and 40 min) on the total sugar content of pumpkin pulp processed with HAD **(a)**, VAD **(b)** and CD **(c)**.

Figure S4. Influence of airflow velocity used in HAD (0.5, 1.0 and 1.5 m/s) on the total sugar content of pumpkin pulp subjected to UAE and CE.

Abbreviations

HAD: hot air drying; VAD: vacuum drying; CD: conductive drying; UAE: ultrasound assisted extraction; CE: conventional extraction

Citation: To be added by editorial staff during production.

Academic Editor: Firstname Lastname

Received: date
Accepted: date
Published: date

Publisher's Note: MDPI stays neutral with regard to jurisdictional claims in published maps and institutional affiliations.

Copyright: © 2022 by the authors. Submitted for possible open access publication under the terms and conditions of the Creative Commons Attribution (CC BY) license (https://creativecommons.org/licenses/by/4.0/).

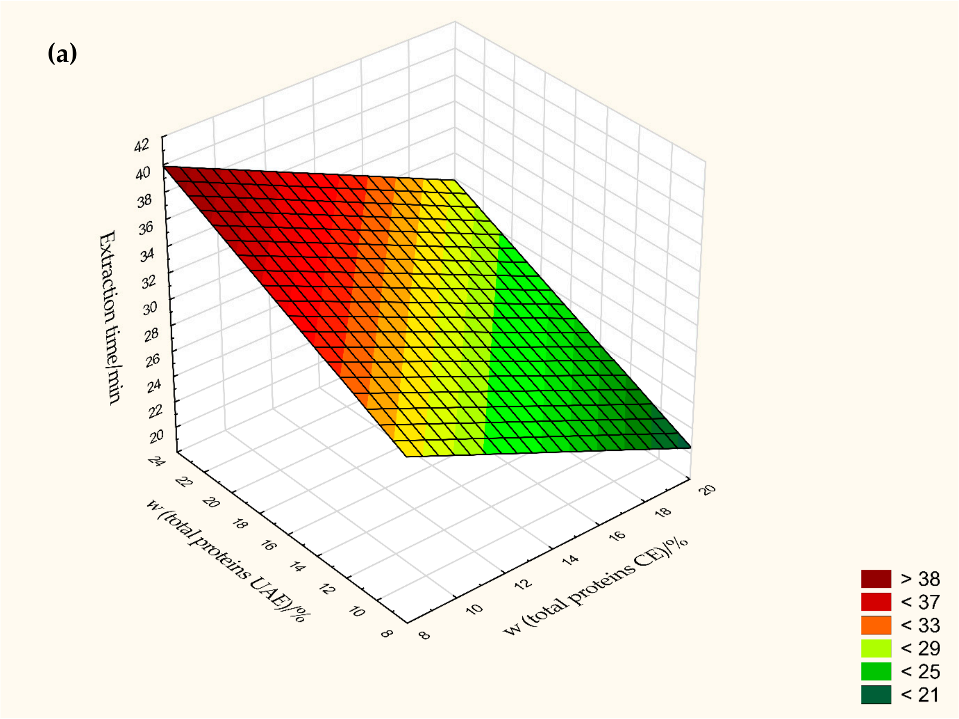
(a)
Extraction time/min
w (total proteins UAE)/%
w (total proteins CE)/%
> 38
< 37
< 33
< 29
< 25
< 21

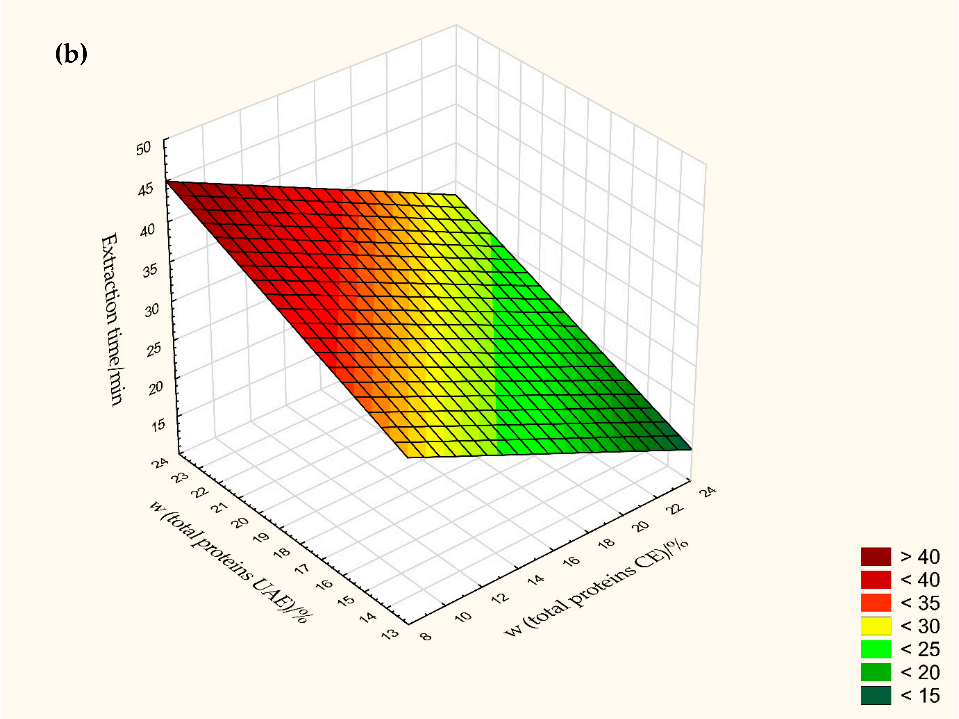
(b)
Extraction time/min
w (total proteins UAE)/%
w (total proteins CE)/%
> 40
< 40
< 35
< 30
< 25
< 20
< 15

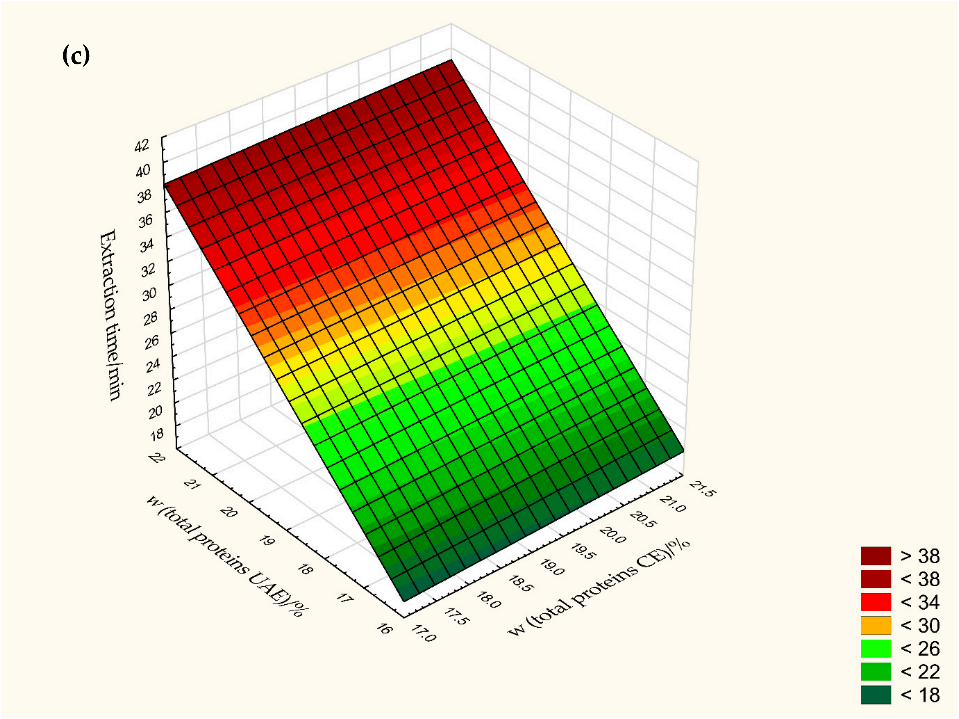

Figure S1. Influence of extraction time in UAE (10, 20 and 40 min) and CE (20 and 40 min) on the total protein content of pumpkin pulp processed with HAD **(a)**, VAD **(b)** and CD **(c)**.

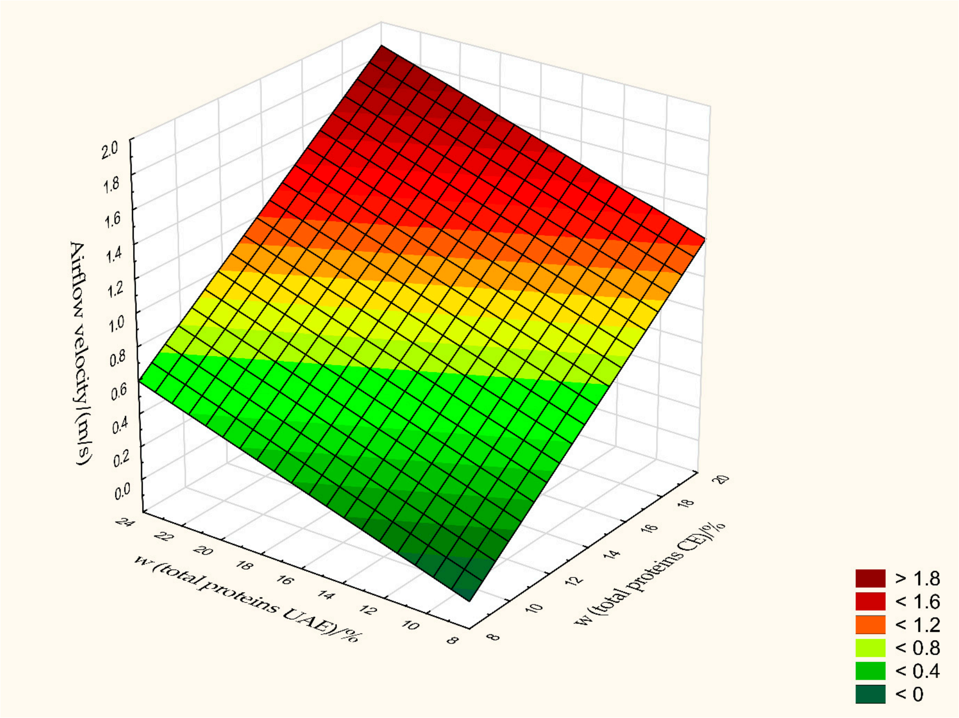

Figure S2. Influence of airflow velocity used in HAD (0.5, 1.0 and 1.5 m/s) on the total protein content of pumpkin pulp subjected to UAE and CE.

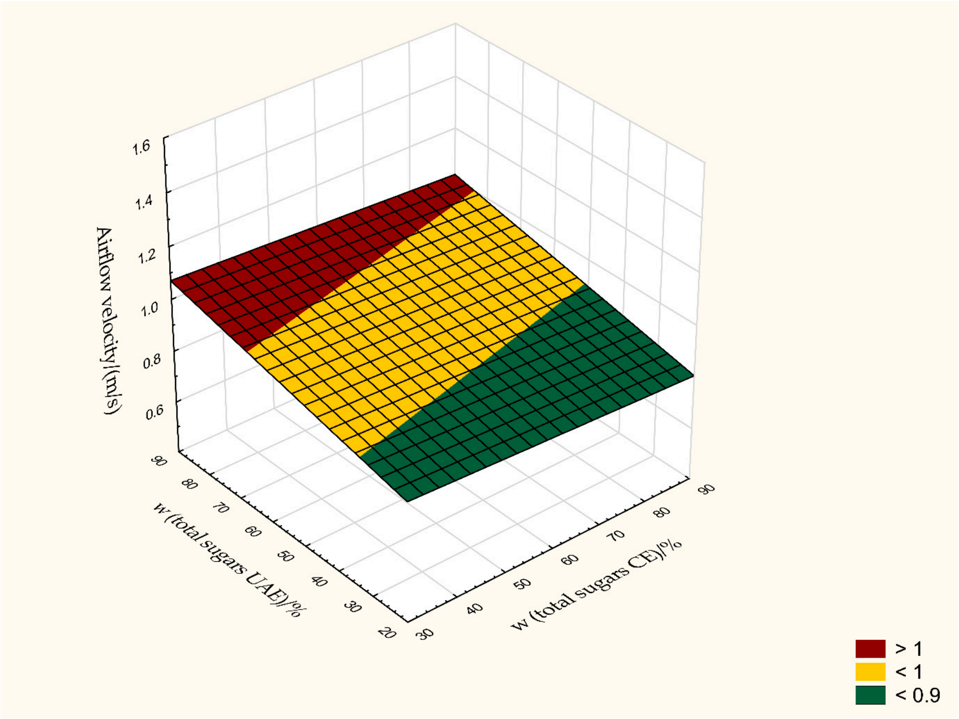

Figure S3. Influence of airflow velocity used in HAD (0.5, 1.0 and 1.5 m/s) on the total sugar content of pumpkin pulp subjected to UAE and CE.

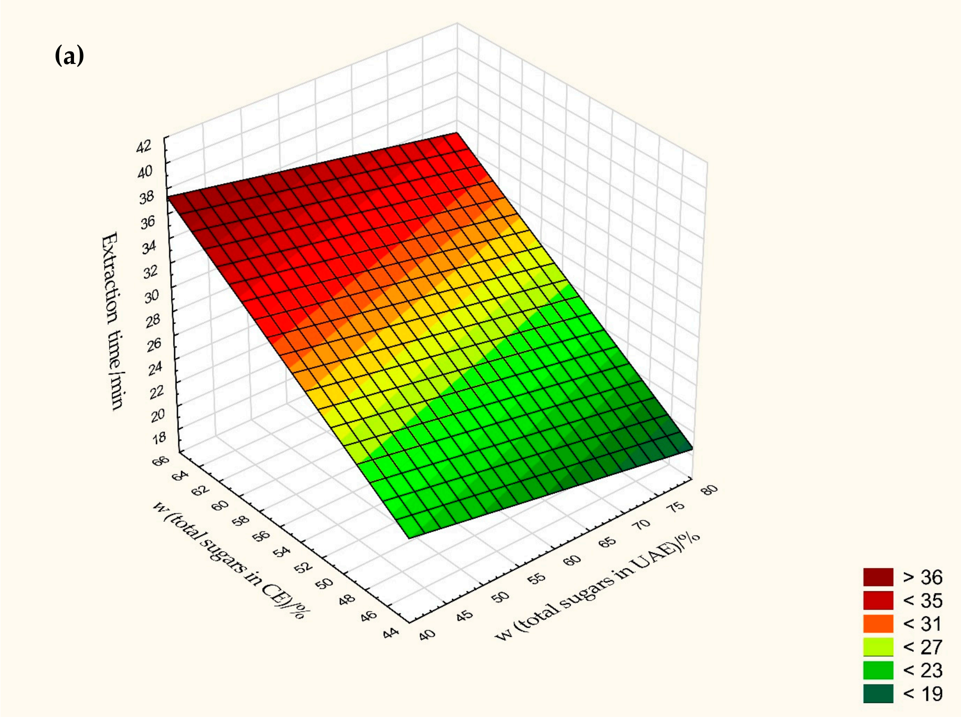

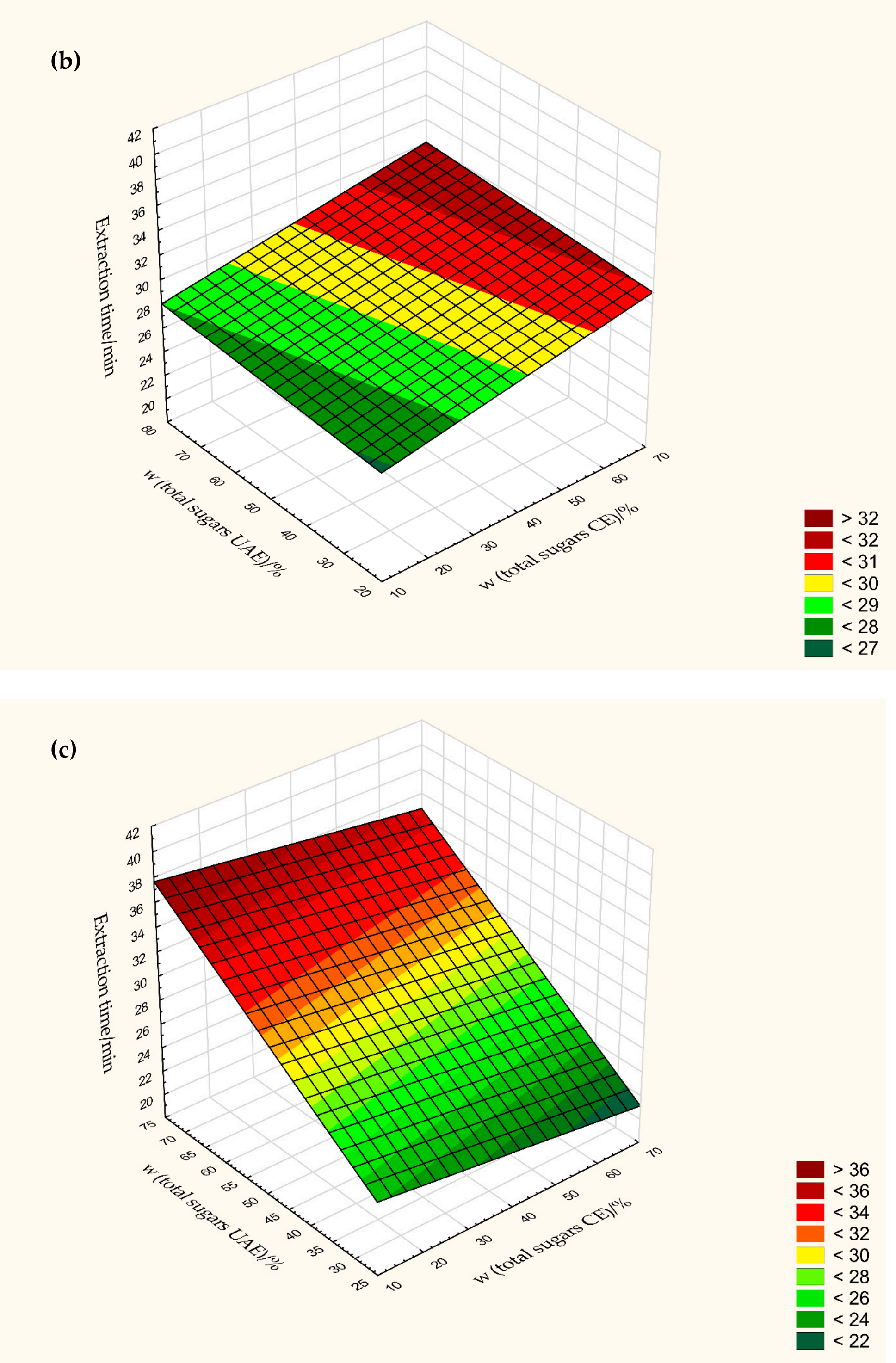

Figure S4. Influence of extraction time in UAE (10, 20 and 40 min) and CE (20 and 40 min) on the total sugar content of pumpkin pulp processed with HAD **(a)**, VAD **(b)** and CD **(c)**.
